# Neural Effects of Gender and Age Interact in Reading

**DOI:** 10.3389/fnins.2019.01115

**Published:** 2019-10-17

**Authors:** William W. Graves, Linsah Coulanges, Hillary Levinson, Olga Boukrina, Lisa L. Conant

**Affiliations:** ^1^Department of Psychology, Rutgers University–Newark, Newark, NJ, United States; ^2^Center for Stroke Rehabilitation Research, Kessler Foundation, West Orange, NJ, United States; ^3^Department of Neurology, Medical College of Wisconsin, Milwaukee, WI, United States

**Keywords:** reading, functional magnetic resonance imaging, gender, age, semantics, orthography, orbitofrontal cortex, cognitive neuroscience

## Abstract

There has been an enduring fascination with the possibility of gender differences in the brain basis of language, yet the evidence has been largely equivocal. Evidence does exist, however, for women being at greater risk than men for developing psychomotor slowing and even Alzheimer disease with advancing age, although this may in part at least be due to women living longer. We examined whether gender, age, or their interaction influenced language-related or more general processes in reading. Reading consists of elements related to language, such as the processing of word sound patterns (phonology) and meanings (semantics), along with the lead-in processes of visual perception and orthographic (visual word form) processing that are specific to reading. To test for any influence of gender and age on either semantic processing or orthography-phonology mapping, we tested for an interaction of these factors on differences between meaningful words and meaningless but pronounceable non-words. We also tested for effects of gender and age on how the number of letters in a word modulates neural activity for reading. This lead-in process presumably relates most to orthography. Behaviorally, reading accuracy declined with age for both men and women, but the decline was steeper for men. Neurally, interactions between gender and age were found exclusively in medial orbitofrontal cortex (mOFC). These factors influenced the word-non-word contrast, but not the parametric effect of number of letters. Men showed increasing activation with age for non-words compared to words. Women showed only slightly decreasing activation with age for novel letter strings. Overall, we found interactive effects of gender and age in the mOFC on the left primarily for novel letter strings, but no such interaction for a contrast that emphasized visual form processing. Thus the interaction of gender with age in the mOFC may relate most to orthography-phonology conversion for unfamiliar letter strings. More generally, this suggests that efforts to investigate effects of gender on language-related tasks may benefit from taking into account age and the type of cognitive process being highlighted.

## Introduction

Responding to the special research topic on examining sex differences in the neural mechanisms of language, we chose to analyze data on reading. Reading interfaces with core language components such as phonology (auditory word forms) and semantic (word meaning) retrieval. It also differs from other language-related tasks such as object naming or speech repetition in that it has distinct lead-in processes of visual perception and orthographic (visual word form) processing (Indefrey and Levelt, 2004). By testing effects of gender (which we use here instead of sex effects because we relied on participant self-identification) together with age for influences on reading, we aim to shed light on the degree to which these factors influence language-related processing such as semantics, or are more related to visual lead-in processes for reading such as orthography. Specifically, to highlight areas responding to semantics, we contrast neural responses to meaningful words with neural responses to meaningless but pronounceable non-words. To highlight areas responding to orthography, we test for neural responses that are parametrically modulated by the number of letters (letter length) in the string being read. These semantic and lead-in responses are then tested for effects of gender and age. As described more fully below, despite an enduring interest in gender effects, they have only inconsistently been reported in language tasks. The current study aims to determine whether gender effects may be reliably seen when taking into account other potentially related effects such as age and specific cognitive demands of the task.

### Gender Effects

A great deal of work has been done on effects of gender in language and reading. Several individual studies point to various types of neural effects of gender in language-related processes such as object naming (Grabowski et al., 2003), visual word learning (Chen et al., 2007), and cerebral laterality of language functions (Mohr et al., 2005; Clements et al., 2006). However, several systematic reviews and meta-analyses have shown little consistent evidence for robust effects of gender either from studies of behavioral performance (Hyde and Linn, 1988) or neuroimaging studies of language-related processes (Ihnen et al., 2009; Kaiser et al., 2009; Wallentin, 2009). If we expand our scope beyond studies of language, however, there may be effects of gender in aging.

### Age Effects

There is general psychomotor slowing in aging (Houx and Jolles, 1993; Keys and White, 2000). This includes tasks related to language and reading such as lexical decision (deciding whether a letter string is a real word) and reading individual words aloud (Balota and Duchek, 1988; Balota et al., 2004). Large-scale studies of reading show similar effects in both younger and older adults for word form-related variables such as number of letters. For semantic variables such as imageability (the degree to which a word elicits a sensory impression), effects are slightly less consistent with age. Higher imageability words were found to be read reliably faster by younger adults when estimates from two different sources (Toglia and Battig, 1978; Cortese and Fugett, 2004) were used. Older adults, on the other hand, only showed reliable effects of imageability on reading aloud for one (Cortese and Fugett, 2004) of the two sources of imageability estimates (Balota et al., 2004). This raises the possibility that effects of age on reading performance may present differently depending on the particular cognitive sub-process involved. Acquiring functional neuroimaging data during reading would allow us to test the possibility that brain regions carrying out semantic and orthographic processing could be modulated differently by age, and perhaps even gender.

### Gender and Age Interaction

While the risk of Alzheimer dementia increases with age for both women and men, the risk has been shown to increase more steeply for women (Fratiglioni et al., 1997; Gao et al., 1998). Even with cases of dementia diagnosis at time point one excluded, scores on the Mini-Mental State Exam (Folstein et al., 1975) were found to worsen faster for women than men with advancing age (Lipnicki et al., 2017). General psychomotor slowing and increased reaction times with increasing age have also been reported to be more pronounced for women (Houx and Jolles, 1993; Der and Deary, 2006). This points to an expected interaction between gender and age, with age-related neural and cognitive changes diverging between genders with increasing age. Regarding language and reading in particular, according to the transmission-deficit model, semantic aspects such as knowledge of word meanings is generally considered to be robust to age-related changes, whereas connections between these meanings and orthographic or phonological representations may be degraded with age (Burke and Shafto, 2004). Overall, there is evidence for an interaction between age and gender in general response speed, yet we found very few studies that specifically examined interactions between age and gender in language-related tasks. Therefore, considering existing evidence for effects of aging on reading and interactions of age with gender on processing speed and risk of cognitive decline, we predict an interaction between gender and age such that any subtle effects of gender on aspects of language will become more evident with increasing age.

Reading offers a particularly useful domain in which to test for the neural and cognitive sources of potential effects of gender and age. This is due to the fact that letter strings can be precisely manipulated to elicit effects thought to arise from different cognitive processes. Semantic processing can be highlighted by contrasting responses to meaningful words with meaningless but pronounceable non-words. Such comparisons have revealed activation for semantic processing in brain areas such as the angular gyrus, posterior cingulate, precuneus, medial prefrontal cortex, and middle temporal gyrus (Binder et al., 2005, 2009).

Reading also involves visual processing of letters and mapping to their corresponding speech sounds. Demands on the initial visual-perceptual step can be modulated by, for example, degrading the quality of the visual presentation or varying the number of letters (length in letters, or letter length) being presented. Interactive effects, where the effect of increasing numbers of letters is enhanced by progressively degrading the words, has been shown in occipito-parietal cortices (Cohen et al., 2008). Manipulating letter length by itself has been shown to elicit greater activation in primary visual cortex with increasing numbers of letters (Mechelli et al., 2000; Wydell et al., 2003; Graves et al., 2010). Here we test the possibility that the enhanced demands on visual and possibly orthographic processing will be potentiated with increasing age, and that this potentiation will differ by gender. Therefore, by contrasting words with non-words, and manipulating letter length in each word, we can examine effects of age and gender on distinct semantic and visual-related aspects of reading.

Here we test three hypotheses concerning possible effects of gender and age in reading. One is that effects of these factors on reading are specifically related to transformations of letter strings into their corresponding sounds or meanings, another is that they are related to visual lead-in processes such as orthography or general visual processing, and a third is that they will influence both language-related processing and more visually related aspects of reading. Making specific predictions about the direction of these potential interactions is complicated by the dearth of studies on how language-related performance differs between men and women with age. Based on the studies noted above, however, it seems that overall processing speed and cognitive abilities are at risk of declining somewhat more for women than men with increasing age. If activation differences between words and non-words differ by gender with increasing age, this would suggest a potentially language-specific (as opposed to visual-processing related) source for interactive effects of age and gender on reading. If, however, there are gender differences with age in the neural activations associated with modulating letter length, this would suggest a visual lead-in source for the interactive effects of gender and age. Alternatively, effects of age and gender may be found for word-non-word differences (lexicality effects) and effects of letter length, suggesting widespread effects of these factors that cut across multiple cognitive domains.

## Materials and Methods

### Participants

Data from the Medical College of Wisconsin (MCW) were pooled across two studies. The first (MCW1, *N* = 18) was published previously (Graves et al., 2010). The second (MCW2, *N* = 15) is from a previously unpublished study of reading aloud. Participants met inclusion criteria for being right handed with no claustrophobia or reported history of neurologic or psychiatric diagnosis. They provided written informed consent according to MCW Institutional Review Board (IRB) guidelines. These data were combined with those from a third study (*N* = 13) performed at the Rutgers University Brain Imaging Center (RUBIC). These participants were generally older and were enrolled as healthy comparisons for a larger study that also involved a separate set of stroke survivors (Boukrina et al., 2015). They provided written informed consent according to Rutgers University IRB guidelines. Data from all three studies provided an overall *N* = 46. Mean age in years was 33.3, max: 78, min: 18, SD: 15.9. The distribution was weighted toward the younger end, with a median of 27.0. The overall sample contained 16 males and 30 females.

### Stimuli

Pooled across all 3 studies, word and pronounceable non-word stimuli (pseudowords, hereafter referred to simply as non-words) were matched for number of letters (length in letters, or letter length), ranging from 2 to 8 letters. They were also matched on a measure of orthotactic typicality. That measure, bigram frequency, is based on the frequency of each word that has the same two-letter combination as the target word in the same position, as used previously (Graves et al., 2010). In study one there were 465 words and 235 non-words. Study two had 300 words and 300 non-words, and study three had 320 words and 165 non-words. Of the 1085 individual word stimuli used across the three studies, 878 of them were unique in that they appeared in only one of the three studies. There were also 700 non-words across the three studies, 491 of which were unique. The MCW2 study contained words and non-words with a slightly greater mean number of letters (4.6, as opposed to 4.4 for the first MCW and RUBIC datasets). The potential influence of this difference is addressed in the Discussion section below.

### Procedure

Word and non-word stimuli were visually presented by projecting onto a rear-projection screen viewed in the scanner by a mirror mounted on the head coil. All studies followed a fast event-related design, which has been shown to be optimal compared to blocked designs for acquiring overt speech responses in the scanner (Birn et al., 2004; Grabowski et al., 2006; Mehta et al., 2006; Soltysik and Hyde, 2006). For MCW1, all the word stimuli were presented first, in randomized order, followed by all non-word stimuli, also in randomized order. For MCW2, all word and non-word stimuli were intermixed and fully randomized. Data from the third study were acquired at RUBIC similarly to MCW1. All the word stimuli were presented first in randomized order, followed by the non-words in randomized order. No stimuli were repeated within a participant session. All participants were instructed to read each letter string aloud as quickly and accurately as possible. Overt speech responses for reading aloud were recorded using a fiber optic microphone connected to a signal processing box. This equipment implemented hard-coded algorithms for real-time dampening of scanner noise to reveal intelligible speech responses.

### Image Acquisition

The MCW1 data were acquired as described previously (Graves et al., 2010). Briefly, participants were scanned using a 3 T GE Excite system with an 8 channel radio frequency head coil. High resolution (0.94 × 0.94 × 1.00 mm) T1-weighted anatomical brain images were acquired using a spoiled-gradient-echo (SPGR) sequence. Functional scans were acquired using a gradient-echo, echoplanar imaging (EPI) sequence in a time series where a whole-brain volume was collected every 2 s (TR = 2) and an echo time (TE) of 25 ms. Each volume consisted of voxels with 3.0 × 3.0 mm in-plane resolution and 2.5 mm thick slices with a 0.5 mm gap between each of the 32 interleaved axial slices. The data acquisition for MCW2 was essentially identical to MCW1, except that the datasets were acquired using a 3T GE 750 scanner with a 32 channel head coil.

Data from RUBIC were collected on a 3T Siemens Magnetom TrioTim Scanner with a 12 channel head coil. T1 high-resolution anatomical brain scans were collected for each subject, using a gradient echo sequence, with a TR of 1900 ms and a TE of 2.52 ms (matrix = 256 × 256 voxels, 176 contiguous 1 mm axial slices, field of view (FOV) = 256 mm, flip angle = 9). Blood Oxygen Level Dependent (BOLD) data were collected using a gradient-echo echoplanar imaging (EPI) sequence (TR = 2000 ms, TE = 25 ms, FOV = 208 mm, matrix = 64 × 64, flip angle = 77). Whole brain volumes (3.3 × 3.3 × 3.0 mm voxels) were obtained, each consisting of 35 axial slices.

### Data Analysis

The accuracy of each reading aloud response was determined by manual playback of the in-scanner audio recording using the freely available Audacity software (https://www.audacityteam.org). Following procedures documented previously (Graves et al., 2010), responses that were omitted or did not conform to standard American English pronunciations of the written words were coded as incorrect. Judgment of non-word accuracies accounted for the fact that there can sometimes be multiple legitimate pronunciations of non-words. Any questions about acceptability of non-word pronunciations were resolved by consensus among native speakers of American English. Examples of types of erroneous responses included lexicalizations (changing the pronunciation of a non-word to conform to a word), addition, subtraction, or transposition of phonemes, or omitting responses entirely. To analyze these accuracy data in terms of subject-level differences in gender and age, we first performed separate multiple linear regression analyses per subject, with reading aloud accuracy as the dependent variable. The individual regression models contained two explanatory/predictor variables: A dummy variable for whether the letter string was a word or non-word (lexicality), and number of letters. We then performed a second-level regression on the mean accuracy per subject, where the seven subject-level explanatory variables were the previously estimated effects of (1) lexicality and (2) number of letters (both derived from the subject-level regression models of response accuracy just described), along with (3) age, (4) gender, (5) an age by gender interaction, (6) and a lexicality by age by gender interaction (corresponding to the interaction of age and gender on the lexicality contrast described below for the neural data), and (7) a dummy coded variable for study/scanner type, as also described below for the neural data. This second-level regression was performed as a non-parametric rank-based procedure so as to be robust to potential outliers, as implemented in the R statistical software package, Rfit (Kloke and McKean, 2012).

Image analyses: Regression analysis using ordinary least squares was carried out using the AFNI program 3dDeconvolve (Cox, 1996). The regression model for analyzing individual subject data included the following regressors of no interest: 6 motion parameters, one for each of three possible directions of rotation and displacement; a low-order polynomial to model low-frequency drift in signal, and mean signal from the lateral ventricles to model non-physiological signal fluctuations within the brain. The regressors of interest were: a binary regressor with a 1 for each trial in which a word was read aloud correctly, otherwise zero; a binary regressor for each correctly read non-word trial; a binary regressor for each trial in which an incorrect response or no response was given; a parametric regressor for number of letters for each word; a parametric regressor for number of letters for each non-word. The parametric regressors were z-score normalized to avoid introducing multi-collinearity with the binary regressors, as performed previously (Graves et al., 2010).

Individual participant results were non-linearly warped into a standard atlas space (Talairach and Tournoux, 1988) using the AFNI program 3dQwarp. These beta-weight images were smoothed with a 5 mm full-width half maximum (FWHM) kernel and averaged for group-level inference. The word-non-word contrast was performed using a two-tailed *t*-test at each voxel, while the parametric effects of word and non-word letter lengths were tested against a null hypothesis of no effect. The group-level program 3dttest++ was used to perform multiple linear regression to test for effects of participant gender (coded as 0 for male and 1 for female), age, the multiplicative interaction of gender and age, and two columns representing a binary covariate of no interest for the model of scanner with which the data were acquired (0 0 = GE Excite, 1 0 = GE 750, 0 1 = Siemens Trio). Mapwise correction for multiple comparisons was then carried out as recommended by Cox et al. (2017) using the “-Clustsim” option in the AFNI program 3dttest++. This option empirically simulates a null distribution that accounts for degree of image smoothness and auto-correlated noise. A voxel-level threshold of *p* < 0.001 and a cluster-level threshold of 664 μl (mm^3^) was applied to the word-non-word contrast for an overall corrected *p* < 0.05. Similarly, for the parametric effect of letter length, a voxel-level threshold of *p* < 0.001 and a cluster-level threshold of 1039 μl was applied for an overall corrected *p* < 0.05.

Note that the procedure just described was implemented using the latest version of the “-Clustsim” option in the AFNI program 3dttest++. This was updated to address the concerns raised by Eklund et al. (2016). Specifically, running 3dttest++ with -Clustsim calculates the smoothness of the data empirically, since the actual smoothness is nearly always greater than the applied smoothness. This empirically estimated smoothness is also used in the updated approach AFNI takes to calculating the auto-correlation function. This function has a mixed Gaussian plus mono-exponential form, which has heavier tails than the Gaussian model. These elements are key to generating an empirical null distribution, which was calculated by simulating 10,000 3D null results. The voxel-wise *p*-value threshold (in our case, *p* < 0.001) was then cross-referenced against the null distributions to give a cluster extent threshold correction to a nominal alpha value of, in our case, less than 0.05. A more detailed documentation of this process can be found in Cox et al. (2017).

## Results

### Behavioral Results

**FIGURE 1:**
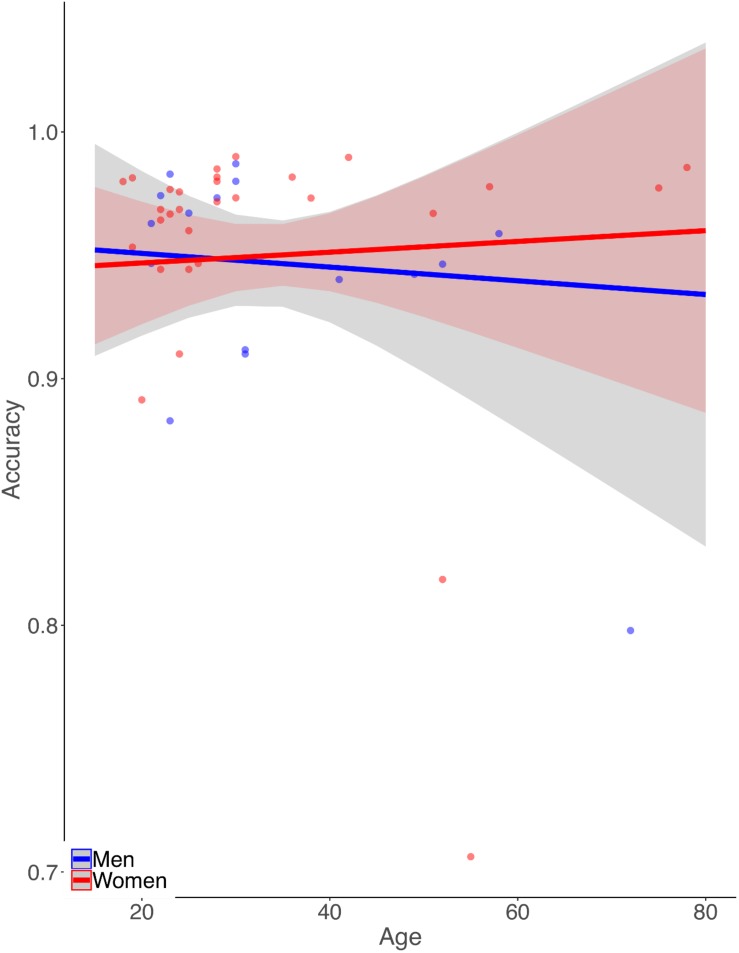
Illustration of the pattern of results from the rank-based multiple linear regression analysis of reading aloud accuracy. Dots represent mean accuracies for individual participants. Accuracy declines more with age for men than for women.

Simple zero-order correlation of accuracy with age showed that on average participants had significantly decreasing accuracy with increasing age (*r* = −0.30, *p* < 0.05). Rank regression revealed an interaction, however, in that women showed a less steep decline in accuracy with age than did men (Figure 1, 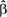 = 0.0013, *p* < 0.01). This effect was small but reliable. Because the mean length of the letter strings was slightly greater for the MCW2 study, we re-analyzed the accuracy data using the same procedures as above but with MCW2 excluded. This was an additional check beyond coding the study separately in the original analysis. The same overall pattern was obtained, with only slightly changed beta weight estimates. Again by zero-order correlation there was decreasing accuracy with age, but with fewer data points the relationship no longer reached statistical reliability (*r* = −0.28, *p* = 0.12). Similarly, rank regression revealed an interaction between effects of gender and age in the same direction as for the full dataset, but with fewer data points no longer reached statistical reliability (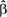 = 0.0013, *p* = 0.07).

### Neuroimaging Results

#### Words – Non-words

**FIGURE 2:**
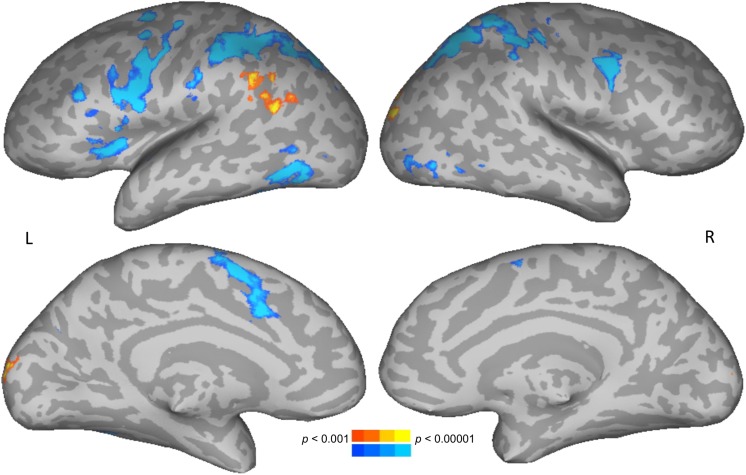
Direct contrast of words (warm colors) compared to non-words (cool colors). Upper row shows lateral views, lower row shows medial views. L, **left**; R, **right**.

To test for brain areas likely to be engaged in semantic processing, reading meaningful words was compared to reading meaningless but pronounceable non-words. Areas more active for words than non-words included the left posterior middle temporal and angular gyri, and bilateral occipital cortices. Areas more active for non-words than words included the bilateral intraparietal sulcus, precentral gyrus, along with left insula and supplementary motor area (Figure 2 and Table 1).

**TABLE 1:** Talairach coordinates, volumes, and absolute value of *z*-scores for the peaks of each cluster showing significant effects related to the words – non-words contrast.

**Location of cluster**	**Cluster size (mm^3^)**	***X***	***Y***	***Z***	***z*-score**
**Words>non-words**					
L posterior middle temporal gyrus	1325	−50	−56	19	4.44
R lateral occipital cortex	1043	25	−89	15	4.79
L angular gyrus	715	−53	−49	35	4.21
L cuneus	700	−6	−97	10	4.54
**Non-words>Words**					
L intraparietal sulcus	11941	−38	−38	39	6.41
L precentral gyrus	10982	−50	2	36	6.82
R intraparietal sulcus	9154	25	−53	47	6.83
L posterior inferior temporal gyrus	3000	−43	−63	−1	4.75
L supplementary motor area	2869	−2	3	54	5.40
R precentral gyrus	2639	44	1	30	5.69
L inferior frontal sulcus	1016	−44	23	27	5.16
**Age effect on words – non-words contrast**					
L medial orbitofrontal cortex	1014	−9	32	−18	6.45
**Interaction of age and gender on words – non-words contrast**					
L medial orbitofrontal cortex	710	−9	32	−18	6.13

*L, left; R, right.*

**FIGURE 3:**
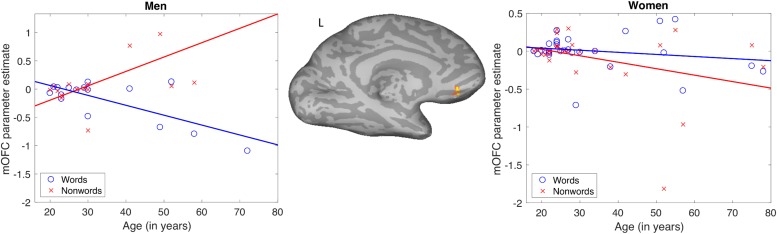
Pattern of significant interaction between gender and age modulating the word-nonword contrast in the left medial orbitofrontal cortex.

The results of the words-non-words contrast were modulated by age in the left medial orbitofrontal cortex (mOFC), where increasing age was associated with decreasing activation. No main effects of gender on this contrast were found. There was, however, an interaction of age and gender. This interaction modulated the word-non-word contrast in essentially the same left mOFC location as did age alone (Table 1). The pattern of the interaction is shown in Figure 3. Women showed no apparent change in activation for words with increasing age, but they did show decreasing activation for non-words with age. Men, by contrast, showed with advancing age a decreasing activation for words, along with increasing activation for non-words.

As also noted above for the behavioral data, a concern with combining data across the three studies included here is that one of them (MCW2) had fully randomized presentation of words and non-words, while in the other two all words were shown first, followed by all non-words. To ensure that the pattern of results seen here was not due to including a study with a different presentation scheme, we conducted a re-analysis with MCW2 data excluded. Although the activation no longer survived cluster-correction due to lack of power from exclusion of the 15-participant dataset, the interaction effects in the mOFC followed the same pattern as for the full dataset and met the voxel-wise threshold of *p* < 0.001 (shown in the Supplementary Figure S1).

In light of the behavioral finding that reading aloud performance declines slightly with age in men but not women, we performed follow-up tests to determine if activation across subjects in the mOFC (the datapoints in Figure 3) was related to accuracy of word or non-word reading. For these *post hoc* tests using Pearson correlations, we found that activation for non-words was reliably correlated with accuracy for men (*r* = −0.56, *p* < 0.05) but not for women (*r* = 0.35, *p* = 0.06). No other zero-order correlations were significant. Although this correlation did not survive correction for four comparisons (words and non-words for men and women), direct comparison between the correlation coefficients for non-word activation and accuracy for men and women did reveal a reliable difference (*z* = 2.93, *p* < 0.01). This was done by performing a Fisher r-to-z transform of the correlations and then calculating a z-score for the difference between the two correlations, as implemented in the R software package, “paired.r”. The observation that accuracy for non-words was negatively correlated with activation in the left mOFC for men only, combined with the pattern of activation in this area decreasing with age in women but increasing with age for men, suggests that the age-related changes in activation for this area in men are maladaptive.

#### Letter Length

**FIGURE 4:**
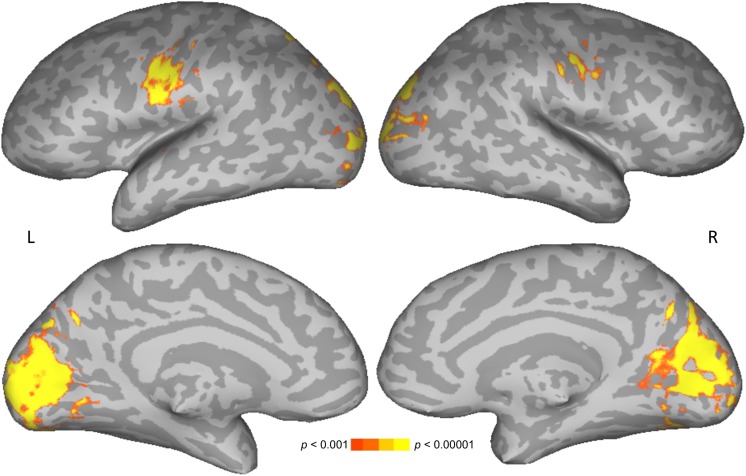
Parametric effect of letter length. Warmer colors indicate significant positive correspondence between neural activation and number of letters.

The main effect of letter length was similar to that reported previously for 18 of the participants in the current 46-participant dataset (Graves et al., 2010), and in other studies (Mechelli et al., 2000; Wydell et al., 2003). Similarities include increasing activation with increasing number of letters in bilateral cuneus and lateral occipital cortices (Figure 4 and Table 2). Unlike in previous studies, activation was also found here for increasing number of letters in bilateral precentral gyrus, presumably related to a correspondence between increasing numbers of letters and increasing demands on articulation for speech in the reading aloud task. No statistically reliable effects of age, gender, or their interaction on parametric effects of letter length were found.

**TABLE 2:** Talairach coordinates, volumes, and absolute value of *z*-scores for the peaks of each cluster showing significant effects of parametric changes in number of letters.

**Location of cluster**	**Cluster size (mm^3^)**	***X***	***Y***	***Z***	***z*-score**
**Parametric effect of number of letters**					
Bilateral cuneus and lateral occipital cortex	42564	−1	−74	16	6.88
L precentral gyrus	4289	−47	−8	28	5.65
R precentral gyrus	1914	47	−4	37	4.79

*L, left; R, right.*

## Discussion

In this study we examined whether gender, age, or their interaction showed specific or general effects in single-word reading aloud. Our finding that gender and age interact to influence the neural contrast between words and non-words suggests that there is a divergence between men and women in how semantic processing changes with age. Effects of letter length, however, were not found to differ with gender, age, or their interaction. Overall, this pattern of results suggests that gender effects emerge from early to late adulthood to influence language-specific processes such as mapping orthographic inputs to phonology, rather than influencing more specifically visual aspects of reading.

Note that effects of gender would have gone largely undetected had we focused exclusively on main effects. We did not find main effects of gender for either the word-non-word contrast or word length. There was, however, a main effect of age on the word-non-word contrast in the left mOFC. This is essentially the same region in which an interaction of gender with age was also found. As reviewed in the Introduction above, our search of the functional neuroimaging literature revealed ample studies examining main effects of gender on language, and some studies examining main effects of age on language. Few studies, however, appeared to specifically test for interactions between age and gender, and we are aware of no others that attempted to narrow down the possible neurocognitive source of such effects.

### Implications of Locating These Findings in the Medial Orbitofrontal Cortex

The current study is, to our knowledge, among the first to detect reading-related changes associated with age and gender in the mOFC. Because modulation of this area was not specifically hypothesized, we can only speculate as to the significance of interactive effects of age and gender on function in this area. Indeed, the mOFC is not generally considered to be part of the canonical reading network in healthy participants (Fiez and Petersen, 1998; Turkeltaub et al., 2002; Jobard et al., 2003; Binder et al., 2005; Graves et al., 2010; Cattinelli et al., 2013; Taylor et al., 2013; Murphy et al., 2019), nor is it typically implicated in the few brain lesion-deficit group studies to date that have investigated the critical neural systems for reading (Ripamonti et al., 2014; Boukrina et al., 2015; Dickens et al., 2019). It is for these reasons that we have taken a cautious approach to interpreting the mOFC findings. One way in which interpretation of activations in this area can be guided is with respect to the fact that men showed steeper decrements in reading accuracy than did women, and their non-word reading accuracy was negatively correlated with their mOFC activations for reading non-words. This suggests that the age-related increase in left mOFC activation for non-words in men is not adaptive. Instead, men may be having to devote more resources to maintaining similar levels of reading as they age, whereas women may be retaining more automaticity.

The overall pattern of relatively stable activation in women compared to men, and their relative preservation of reading accuracy with age, runs counter to the initial pattern we noted from previous literature that women may be more vulnerable to pathological changes with aging such as development of psychomotor slowing and dementia. This prompted us to revisit the issue of women possibly being at greater risk of dementia. Indeed, some relatively recent studies have shown higher rates of mild cognitive impairment in men at earlier ages (Petersen et al., 2010; Jack et al., 2019). This, combined with evidence of slower metabolic aging of the brain among women (Goyal et al., 2019), suggests that the initial finding of higher incidence of dementia in women might have been confounded with the fact that women have a longer average life expectancy (Pinkhasov et al., 2010). Overall then, gender-specific predictions for age effects may not have been obvious.

The studies just discussed deal with general associations between gender and aging. Few studies have specifically examined interactions between age and gender in language-related tasks. Again with the current results in mind, we re-examined the literature. The handful of relevant studies we found showed somewhat mixed results, but generally pointed toward relatively less decline in language measures with advancing age for women compared to men. Specifically, men were found to have declining verbal memory with age, whereas women were not, although that study did not compare men and women directly (Gur and Gur, 2002). Other studies found a non-significant trend toward greater declines for men than women in category-specific word production (category fluency; McCarrey et al., 2016), and significantly greater declines for men compared to women in diversity of syntactic structure use and increasing speech disfluencies (Moscoso et al., 2017).

Whether the changes in activation patterns with age and gender shown here relate to pathological changes in the mOFC is a question the current study is not be well positioned to address. We had a limited number of participants at the older end of the age range, and no participants reported concerns with memory or performance. Changes in mOFC are, however, consistent with studies showing it to be among several regions that show strong correlations between age and reductions in volume and cortical thickness (Storsve et al., 2014). It is also a major area that shows pathology with the advance of Alzheimer disease, including both amyloid beta deposition (Buckner et al., 2009) and neurofibrillary tangles (Van Hoesen et al., 2000), suggesting the mOFC may be particularly sensitive to age-related changes. Studies of the morphology of the OFC have also reported gender differences, particularly in the medial as opposed to more lateral regions of the OFC (Wood et al., 2008; Núñez et al., 2018).

In terms of possible task-related interpretations of functional effects in the mOFC, activation in this area has been associated with decision making (Rolls and Grabenhorst, 2008), and in particular with making choices in incompletely specified situations (Elliott et al., 2000). This may be relevant to pronouncing many of the stimuli used in the current study, which included unfamiliar words and non-words that are entirely novel letter strings where one may need to choose among multiple possible pronunciations. To the extent that non-word pronunciations are rule-based, a study that examined age-related changes in activation for a task involving matching words based on rules may be relevant. When words were matched based on atypical compared to typical rules, older participants showed more activation than younger participants in several areas including mOFC (Methqal et al., 2017). Considering the current results in light of these previous studies suggests the functional changes seen in the mOFC may be related to compensation for age-related changes in the neural circuitry involved in selecting among possible pronunciations for a given letter string.

Additionally, gender effects may come into play to the extent that selecting a proper pronunciation involves decision making. Gender differences have in fact been previously reported in the mOFC when having to make decisions in the face of uncertainty. Specifically using the Iowa gambling task, lateralization differences between men and women were found for effects of lesions that included the mOFC (Tranel et al., 2005). A functional neuroimaging study of the same task also found that signal in the mOFC showed differences by gender (Bolla et al., 2004).

#### Potential Limitations

Most of the potential limitations to the current study relate to the need to combine data across multiple datasets in order to have sufficient power to detect effects of an interaction between gender and age. This led to a slight imbalance in the mean number of letters across studies. Specifically, the second dataset collected at MCW had stimuli with slightly more letters on average than the other two datasets. Note that the matching of length between words and non-words across studies was not affected. The MCW2 study was also different in that it was the only one in which word and non-word presentations were fully randomized. In the other two studies, all the words appeared first, with word order randomized, followed by non-words with non-word order randomized. Having slightly longer stimuli and a slightly different design in one study risks confounding study membership with letter length and effects of study design. We directly addressed the possibility of these confounds in the behavioral and neuroimaging data in two ways. The first was by including a covariate to account for differences between studies in the regression analyses, and the second was by re-analyzing the data after removing MCW2. As reported in the Results section above, the pattern of behavioral results after removal of MCW2 was unchanged. Similarly, the pattern of neuroimaging results was also unchanged. Additionally, we note that the gender distribution was imbalanced, such that there were nearly twice as many females as males overall. The exact effects of such a gender imbalance on the results are unclear. One implication, however, relates to the fact that we are not claiming that mOFC is the only structure that could show an interaction of gender and age in reading. Indeed, it is possible that a larger dataset with more participants and more balanced distributions of gender and age would enable us to detect interactions of these factors in other brain regions as well.

Overall, this study of single-word reading aloud has replicated some of the standard findings for the direct contrast between meaningful words and meaningless but pronounceable non-words, thereby highlighting differences in the neural systems involved in retrieving word meaning compared to decoding letter combinations into speech sounds. A parametric analysis of letter length also replicated previous findings on the visual lead-in processes for reading in bilateral primary visual cortices. Main effects of gender were not found, but interactions of gender with age were found exclusively in mOFC for the lexical effect. While specific interpretations of the mOFC finding await confirmation and clarification in future studies, a major contribution of the current study is the demonstration that the previously reported inconsistent effects of gender in language tasks may reflect the need to consider gender in the context of other interacting factors such as age and specific cognitive task demands.

## Data Availability Statement

The datasets generated for this study are available on request to the corresponding author.

## Ethics Statement

The studies involving human participants were reviewed and approved by The Medical College of Wisconsin Institutional Review Board and The Rutgers University Arts and Sciences Institutional Review Board. The participants provided their written informed consent to participate in this study.

## Author Contributions

WG, LLC, and OB designed the studies. WG, LC, and OB analyzed the data. WG drafted the manuscript. All authors contributed to collecting the data, editing, and revising the manuscript.

## Conflict of Interest

The authors declare that the research was conducted in the absence of any commercial or financial relationships that could be construed as a potential conflict of interest.
