# Supplementary material for: Neural Effects of Gender and Age Interact in Reading
Source: Front Neurosci. 2019 Oct 17;13:1115. doi: 10.3389/fnins.2019.01115 (PMC6812500; doi:10.3389/fnins.2019.01115)
Supplement: Supplementary file 1 [file Data_Sheet_1.PDF]

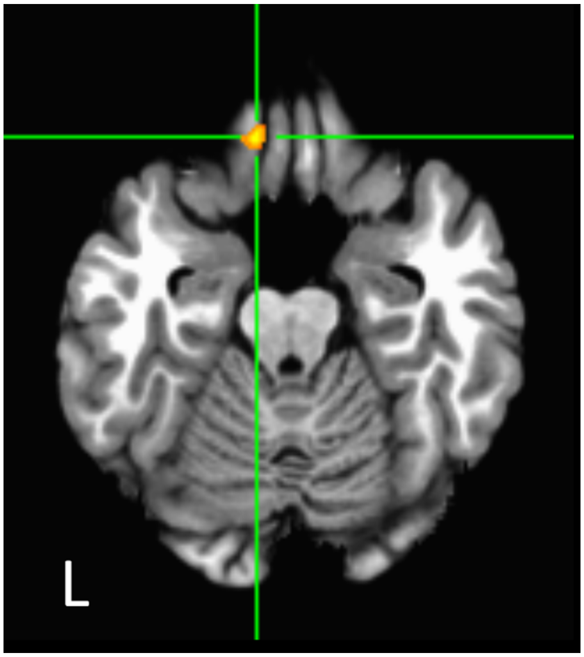

Supplementary Figure: Brain area (medial orbitofrontal cortex) showing and interaction of gender and age on the word-nonword contrast, after exclusion of the MCW2 study. Activation is thresholded at a voxel-level $p < 0.001$. The location displayed in the center of the green crosshairs (-9, 32, -18) is the location of the peak for the same interaction effect in the full dataset. Although without the MCW2 data it no longer survives cluster correction for multiple comparisons, this interaction is in the same direction as shown in Figure 3. L = left.
